# Sperm-Egg Fusion: A Molecular Enigma of Mammalian Reproduction

**DOI:** 10.3390/ijms150610652

**Published:** 2014-06-13

**Authors:** Karolina Klinovska, Natasa Sebkova, Katerina Dvorakova-Hortova

**Affiliations:** 1 BIOCEV Group, Department of Zoology, Charles University in Prague, Vinicna 7, Prague 2 128 44, Czech Republic; E-Mails: K.Klinovska@seznam.cz (K.K.); natasa.sebkova@natur.cuni.cz (N.S.); 2 Cell Biology, Faculty of Science, Charles University in Prague, Vinicna 7, Prague 2 128 44, Czech Republic

**Keywords:** gamete fusion, IZUMO1, Juno, tetraspanins, integrins

## Abstract

The mechanism of gamete fusion remains largely unknown on a molecular level despite its indisputable significance. Only a few of the molecules required for membrane interaction are known, among them IZUMO1, which is present on sperm, tetraspanin CD9, which is present on the egg, and the newly found oolema protein named Juno. A concept of a large multiprotein complex on both membranes forming fusion machinery has recently emerged. The Juno and IZUMO1, up to present, is the only known extracellular receptor pair in the process of fertilization, thus, facilitating the essential binding of gametes. However, neither IZUMO1 nor Juno appears to be the fusogenic protein. At the same time, the tetraspanin is expected to play a role in organizing the egg membrane order and to interact laterally with other factors. This review summarizes, to present, the known molecules involved in the process of sperm-egg fusion. The complexity and expected redundancy of the involved factors makes the process an intricate and still poorly understood mechanism, which is difficult to comprehend in its full distinction.

## 1. Introduction

Fertilization is a multistep and complex process culminating in a merger of gamete membranes, cytoplasmic unity and fusion of genomes, initiating the development of a new individual. Even though membrane fusion is a key event in this process, there is still very little known about its mechanism or the molecules involved. Fusion shows less distinct species-specificity than do the preceding steps in fertilization, like *zona pellucida*-sperm interaction [1], which suggests that the mechanism and molecules involved in membrane fusion are more conserved. During recent years, efforts have been made towards the identification of the molecular players and their function, and several molecules on the egg or the sperm side have been found to be essential or nearly essential. Although the concept of multiprotein complexes on both membranes has been accepted in recent years, the first known molecules of direct interaction in mammalian fertilization have only recently been discovered [2]. 

The only truly essential player on the sperms’ side known to date is the protein IZUMO1, in which knock-out is completely infertile due to impaired fusion [3]. Several proteins have been found to interact laterally with IZUMO1 in the membrane [4]. It has just been found that its prime-binding partner on the oolema is a folate receptor 4 named Juno, which represents the first discovered indisputably vital molecule on the side of the mammalian egg [2]. 

## 2. Fusion as a Crucial Biological Event

Membrane fusion is one of the most fundamental processes in multicellular organisms, enabling a wide range of actions, such as sexual reproduction, vesicular trafficking, immune reactions, and neurotransmission. While this study concentrates on molecules participating in gamete fusion, knowledge of the general mechanism in a different context could be truly helpful. Membrane fusion has been extensively studied for many years, yet the overall picture of the mechanism is far from complete. The mixing of two phospholipid bilayers occurs in three contexts—virus-cell fusion, intracellular vesicle fusion and cell-cell fusion. Although virus-cell and vesicle fusion are relatively well known (reviewed by [5]), cell-cell fusion mechanism remains surprisingly unknown despite its physiological importance. However, it is expected that the mechanics of all three processes should be at least partially similar despite the differences between conditions in which they take place.

There have been many attempts to divide the fusion process into stages, to make the comparisons easier. They vary among each other, but generally it can be said that the process requires the gaining of cellular competence to fuse, membrane recognition and attachment, induction, and activation of the fusion-associated membrane molecule, apposition, and finally lipid bilayers mixing [6,7]. In different systems the target specificity is ensured in different stages-either membrane recognition, or induction (sperm unable to fuse with an egg still binds to it, [2]). The conceptual framework in the field assumes specific proteins on the membrane that are essential for fusion to be either involved in attachment, or mediate the merging of the cell membranes [7].

Regarding the attachment, in many virus-cell fusion systems multiple proteins participate in a single virus-cell attachment event, facilitating a complex interaction occurring in a limited time frame. This fusion machinery often comprises of adhesion domains or carbohydrate moieties on membrane proteins [8]. It is predicted that cell-cell attachment proteins would share these characteristics, as the immunoglobulin (Ig) superfamily members involved in Drosophila myoblast fusion do for example [9]. Each of these proteins contains several Ig-like domains, which are well-defined cell-cell adhesive domains. 

The lone process of fusion is mediated by cell fusion proteins (fusogens) bringing the membranes closer together and mediating the mixing of bilayers. Upon receiving the induction signal these molecules, linking the inter-membrane space, irreversibly fold back on themselves in a hinge-like motion and draw the membranes very close together, enabling the two lipid bilayers to mix [10]. In well-investigated systems, several molecules have been identified as *bona fide* fusogens, *i.e.*, in Gp41 in viral fusion [11,12] or SNARE proteins in synaptic vesicles fusion [13,14].

In mammals, one family of well-defined fusogens named syncitins has been reported. This family includes proteins derived from endogenous retroviruses related to the HIV (human immunodeficiency virus) Gp41 envelope glycoprotein, and function during the formation of the syncitial trophoblast that is essential for mouse placentation [15]. Syncitins were proved to be *bona fide* fusogens, as they induce cell-cell fusion in different cell lines in a receptor-dependent manner, with disulphide bridge-forming motifs essential for their fusogenic activities [16].

The fusogens in other systems and species are being intensively hunted. The difficulties in this field are mainly caused by the fact that based on work on viral fusogens, it seems that the overall structure rather than the primary sequence is conserved [17], and the fact that cell-cell fusion is believed to be restricted to specific cell types, which are often complicated to work with.

## 3. Interaction of Gametes Culminating in Fusion

Despite the amazing variety of organisms, it still takes two to tango in sexual reproduction—sperm and egg meet and fuse to ensure the mixing of genetic material and the development of a new unique individual. On the way, gametes (especially the sperm) undergo series of events changing their morphology, structure and functionality, only to allow them to recognize each other and fuse. Eggs acquire the competence to fuse with sperm once they are at least 20 μm in diameter while still arrested in prophase of the first meiotic division [18]. Sperm experience a great transformation to become a fertilization-competent during its passage through the female reproductive tract, with the capacitation and acrosome reaction changing its motility, physiology, and molecular membrane structure without which the sperm fails to pass on its precious genetic cargo.

Capacitation is the first step to render sperm capable of interaction with the egg. It is basically a functional maturation of the sperm, involving an increase in membrane fluidity due to cholesterol efflux, changes in sperm membrane potential, increased tyrosine phosphorylation and induction of hyperactivation [19]. It is followed by the acrosome reaction—fusion of the plasma and outer acrosome membranes, exposing the inner acrosome membrane and releasing the acrosomal content. It can be triggered by multiple factors—contact with ZP (*zona pellucida*), progesterone concentration or even spontaneously—suggesting that timing of this essential process is redundant and the different time of onset in different population of sperm may play a role in sperm competition [20]. The exact combination of causes and effects is not clear, however, it is well known that the acrosome reacted sperm penetrates the ZP, enters the perivitelline space and is able of fuse with the oolema [21]. The sperm cells that did not undergo this process bind to the egg, but are incapable of fusing with it, which indicates that the essential factors on the sperm membrane are either exposed or modified by this massive acrosomal exocytosis [22].

The fusion site is specific on both gametes, which leads us to believe that there are topologically unique protein populations or lipid organization sites with the distinct membrane morphology required for fusion [6]. The sperm membrane overlying the acrosome, which does not take part in the acrosome reaction, is called the equatorial region, and the sperm-egg fusion is long believed to be initiated in this region [1]. The surface of egg plasma membrane can be divided into two parts: the microvillar-free smooth region, which overlays the meiotic spindle, and the microvillar protrusions-rich region, covering the rest of the egg, forming a dome shaped structure antipodal to eccentric nucleus. Gamete fusion occurs predominantly [23] or exclusively [24] in the microvillar-rich region.

When the two membranes are merged, creating a new zygote membrane, the inner acrosomal membrane, forming the anterior of the sperm head, is excluded from the merger. It fuses with a small patch of the oolema and forms a separate detached hybrid vesicle in the cytoplasm, in a process described as pseudo-phagocytotic-like [25].

Despite great efforts, the molecular basis of the gamete interaction is still poorly understood. During the course of research history, there have been many shifts in paradigms, completely dismissing the previous view and building a new one on recent discoveries. Naturally, this has been made possible by the advances in technology. Monoclonal antibodies, *in vitro* fertilization and particularly knock-out organisms with no expression of a specific molecule are methods which have changed our understanding the most, and now represent the fundamental technologies in the field. For example, the method of producing knock-out mice strains has shaken the existing belief that integrins are the most important adhesion and fusion molecules on the egg. Knockouts have proven that there is no integrin essential for fusion, which occurs even when integrins are not expressed at all [26]. Thanks to this method, the only truly essential fusion/binding factors known, thus far, remain sperm IZUMO [2] and egg Juno [2], as well as CD9.

## 4. Identified Players in Gamete Fusion in Mouse

In the last couple of years, a completely new picture of fusion machinery is emerging thanks to a protracted unraveling of the molecules involved. Even though our comprehension is far from complete, some molecules are established as essential, nearly essential, or associated with the essential factors, forming an intricate and partly redundant system securing the process of fertilization.

### 4.1. Essential Molecules on the Sperm Side

#### 4.1.1. SLLP1 (Sperm Lyzozyme-Like Acrosomal Protein)

In 2005, Herrero *et al.* [27] discovered the mouse sperm lyzozyme-like acrosomal protein (SLLP1) that relocates into the equatorial segment after the acrosome reaction. It was proposed to play a role in gamete interaction, which was proved by *in vitro* fertilization assay, where the specific antibody against SLLP1 blocked both fertilization and binding. Receptor sites for this protein are found in the microvillar region of the egg and in the perivitelline space, which is in agreement with localization of CD9 [28]. The binding partner of SLLP1 was however found to be SAS1B (Sperm Acrosomal SLLP1 Binding), a specific oolemal metalloprotease [29].

#### 4.1.2. IZUMO1

At the beginning of IZUMO1 discovery, the monoclonal anti-mouse antibody against an unknown antigen on the sperm surface (inhibiting the fusion process both *in vivo* and *in vitro)* was characterized through screening of anti-sperm monoclonal antibodies [30]. This antibody was named OBF13 and its corresponding antigen was not identified for many years. In 2005, Inoue *et al.* [2] characterized this protein by 2D gel electrophoresis, immunoblotting, and liquid chromatography-tandem mass spectrometry analysis, and named it IZUMO after a Japanese shrine dedicated to marriage. The question whether IZUMO1 functions as a truly essential factor in fertilization could have been answered only by generating *Izumo1*-deficient mice by homologous recombination. *Izumo*^−/−^ mice were found healthy and without any developmental abnormalities, but as expected the males were sterile despite normal mating behavior. The sperm penetrated the ZP without any problems but failed to fuse with eggs, resulting in accumulation of sperm in the perivitelline space of the egg. *Izumo*^−/−^ sperm defect is limited to fusion ability, as proven by an injection of the sperm into wild type eggs, which led to normal implantation, to full-term development of the offspring at normal ratio with an ability to reproduce.

IZUMO was found to belong to an immunoglobulin superfamily of type I membrane proteins with one extracellular immunoglobulin (Ig) domain and one *N*-terminal domain. The superfamily consists of four proteins, coded with numbers 1 to 4, showing a significant homology in the *N*-terminal domain, hence known as “IZUMO domain”. IZUMO1 (originally described by Inoue’s group), 2 and 3 are transmembrane proteins expressed only in the testis, whereas IZUMO4 is soluble and expressed in the testis and other tissues [3].

The fusion-indispensable IZUMO1 is not expressed in the same place on the sperm during its course through the female reproductive tract and fertilization process, especially during acrosome reaction. Sperm can be divided into three groups depending on their acrosomal reaction state and IZUMO1 staining pattern—acrosomal cap, equatorial and whole head. IZUMO1 relocates during acrosome reaction from the anterior part of the sperm head to the sites where the fusion would take place. Since it is said that sperm launches the fusion with an egg at the equatorial segment, either equatorial or whole-head type IZUMO1 can contribute to sperm-egg fusion.

Although IZUMO1 is the only known essential factor in the sperm, and is often described as the primary fusogen of the sperm side, its only functional domain is an immunoglobulin one. The molecule lacks any fusogenic peptide domain or domain resembling fusogenic peptides in other systems like viral penetration or intracellular vesicular trafficking. Therefore, it seems probable that IZUMO1 interacts with associated proteins that directly facilitate the fusion process in a multiprotein complex on the sperm membrane [31]. 

Ongoing research is directed at searching for these associated proteins, as it is predicted that the functional domain would at least share characteristics with other fusogens *per se* and the factor is expected to be essential, and therefore would block the fusion in knock-out systems. Nevertheless, the Ig proteins are well known to function as an antigen receptor, co-receptor and adhesion molecule through interactions. It was therefore expected that IZUMO1 interacts directly with some molecule on the oolema. It has been found that the ligand for IZUMO1 is a folate receptor 4 [2], however, the precise function of the IZUMO1 protein remains to be unraveled, whether it is a regulator of the fusogen, or/and just an adhesion molecule. However, new investigations have shown that a helical dimer of fragments of *N*-terminus domain of IZUMO1 is required for the sperm-egg fusion [32]. Nevertheless, experiments with Cos-7 cells (African Green Monkey Cercopithecus aethiops Fibroblast-like Kidney Cells) expressing the whole IZUMO1 molecule showed that IZUMO1 binds to eggs, but fails to induce fusion. This implies the role of IZUMO1 to be related to membrane interactions, not to their fusion [32].

##### Proteins Associated with IZUMO1

Previously published works have led to an easy assumption that IZUMO1 is a sperm fusogen. However, the protein lacks any fusogenic peptide part or “SNARE” like structure (Soluble NSF (*N*-ethylmaleimide-sensitive fusion protein) Attachment Protein Receptor), as would be expected by findings in other fusion systems of mammalian cells. This opens the possibility that IZUMO1 could be an essential factor in a protein complex that might contain or modulate other fusion molecules. Ellerman *et al.*, 2009 [3] showed that IZUMO1 forms complexes with other proteins on the sperm surface and suggested that its *N*-terminal domain possesses the ability to form dimers. This supports the hypothesis that IZUMO1 is involved in organizing or stabilizing a multiprotein complex essential for the function of the membrane fusion machinery. With this in mind, Inoue *et al.*, 2010 [33], found a protein located on the sperm acrosomal cap that could interact with IZUMO1 and participate in the process of fertilization. The promising protein was identified as ACE3 (Angiotensin Converting Enzyme-3). However, it was found that ACE3 disappears from the membrane after acrosome reaction and its knock-outs have no reproductive disability both *in vivo* and *in vitro*.

#### 4.1.3. Integrins and Their Receptors

Many experiments have initially shown integrins as important agents participating in the process of sperm-egg interaction on the egg side, as was the case of integrin α6β1 [34]. Although originally considered promising, it was later shown through knock-out experiments that α6β1 deficient eggs are fertile in *in vitro* assay [35]. However, these experiments were carried out with wild type sperm and it was shown that α6β1 is expressed on sperm [36]. It may be possible that integrin molecules on sperm, substitute those, which are lacking on the egg surface. This eventuality is supported by the discovery of exosome-like vesicles from the oolemal surface that transfer material to the sperm head and possibly vice versa [37]. The notion of an intricate correlation system containing integrins is supported by a deemed receptor of integrin α6β1-β1 receptor fertilin β on the sperm membrane (also known as a Disintegrin and Metalloprotease2—ADAM2) [38,39]. It appears to enhance the initial adhesion of sperm to the oolema and to increase the sperm attachment rate [40] and mice sperm lacking fertlin β display a defect in sperm-egg membrane adhesion and fusion [41,42,43]. The ADAM protein family appears to be of great importance for the whole process of fertilization. Members like fertilin β, ADAM3 and others form an intricate and complex system of molecules playing a role in sperm migration throughout the oviduct [44] and binding to *zona pellucida* [45].

#### 4.1.4. CD46

There are many factors on sperm associated with integrins that may play a role in the fusogenic machinery, however a redundant role that may be. One of these is CD46, which is expressed in rodents on the acrosomal membrane of sperm [46]. Monoclonal antibodies raised against its ectodomain block binding of human sperm to *zona*-free eggs *in vitro* [47] and so its potential part in the attachment and fusion process has been proposed. The fact that CD46 has been found to interact directly with β1 integrins and indirectly with tetraspanins in human cells [48] only supports the hypothesis. However, knock-out experiments have shown that CD46-deficiant mice are fertile, with accelerated spontaneous acrosome reaction [49] and, thus, the main function of CD46 appears to stabilize the acrosomal membrane [50]. Nevertheless, due to the expected complexity of proteins involved in the attachment machinery, CD46 cannot be ruled out. 

### 4.2. Essential Molecules on the Egg Side

Throughout the long studies of gamete interaction, a few families of proteins have been proposed as binding or fusogenic players on the egg’s side. Many of the previously described proteins were proven to be false leads by current knock-out mouse lines, nevertheless, a couple of candidates are emerging to shed light on the fusogenic machinery on the egg surface.

#### 4.2.1. Tetraspanin Family

The tetraspanin family consists of small (20–50 kD) transmembrane proteins that contain four transmembrane domains (which gave the family its name) with two extracellular loops and wide tissue distribution. Through the larger extracellular loop, the molecule directly or indirectly *cis*-interacts with other membrane proteins as immunoglobulins (Ig), signaling enzymes and integrins [51], and mediate the assembly of structural and functional units called tetraspanin enriched microdomains (TEMs), analogous to microdomain lipid rafts [52]. Tetraspanins also communicate with cytoskeletal and signalling molecules via intracellular domains [53]. Within TEMs, tetraspanins are believed to be primarily the organizers of the network of transmembrane and cytoplasmic effector molecules, such as receptors, fusogens, and signaling proteins, and modulate their function and, thus, regulate many processes. The tetraspanin network is used to execute functions that require multiple intermolecular interactions. It has been reported that some tetraspanin molecules may act as receptors, but so far the examples are few (*i.e.*, [54]). The conservation of tetraspanin genes suggests they appeared early in evolution and perform vital functions.

##### CD9

An important member of the tetraspanin family is the ubiquitously expressed protein CD9. It functions as a regulator of a wide range of processes varying from the fusion of myoblasts [55] and monocytes [56] to cell signaling and adhesion. As expected from such a versatile tetraspanin protein, it interacts laterally with many other molecules, including immunoglobulins [57], other tetraspanins, a subset of integrins, G proteins, or other adhesion molecules [58].

At first, it was reported that the antibody against CD9 reduces the fertilization rate [59]. The discovery that the protein is nearly irreplaceable for fertilization was made by chance serendipitously and simultaneously in three laboratories during research on the effects of CD9 knock-out on the immune system [60,61,62]. It was found that the knock-out mice are healthy, but females have severely reduced fertility. The defect is fusion related, as the wild type sperm penetrates the *zona pellucida*, binds to the oolema but the membranes fail to fuse. The infertility is overcome by intracytoplasmic sperm injection (ISCI), and embryos develop normally. This was a very striking and uncommon knock-out phenotype, as it represents a widely expressed protein, manifesting a very specific, non-redundant effect only in the egg, where other proteins from the same family are also expressed, but unable to replace its function.

As CD9 was, for a long time, thought to be the only known factor to be completely essential on the egg, it led to its exact function being extensively studied. Three theories have been proposed about its function—*cis*-interaction with other membrane proteins, trans-interaction with a sperm receptor or a role in the membrane structure. These hypotheses do not exclude each other and indeed all three can be proven to be correct. 

The second hypothesis about trans-interaction arose from the research of macrophage regulation, where CD9 was found to bind to pregnancy specific glycoprotein 17 (PSG17), a member of immunoglobulin superfamily [63]. The glycoprotein was found to inhibit fusion if added to zona-free eggs [64], but is not discovered on sperm surface. At least two related glycoproteins from the sub-family are expressed in the sperm, but no evidence of their role in sperm-egg fusion has yet been presented [25]. According to new research, CD9 may play a role in sperm-egg binding rather than fusion. CD9-null eggs show reduced ability for strong sperm adhesion [65], and sperm accumulate in the perivitelline space, only transiently binding to the egg surface. This suggests a role in adhesion strengthening [66]. 

The assumption about the CD9 role in membrane order was proven to be correct by researches of microvilli organization and morphology in CD9-defficient eggs. Immunofluorescence shows that CD9 is localized to the microvillar region of the egg [61], which is a specific protrusion, rather than to the planar membrane between them [67]. In knock-out mice, the morphology of the microvilli is altered, as these appear shorter, thicker, and loosely distributed, with the radius of the curvature appearing wider [65]. If we accept the role in membrane order, we can also easily assume that the first hypothesis about cis-interaction is correct, as such a significant role in morphology surely demands cooperation with other membrane proteins.

It was also proposed that exosome-like CD9-containing vesicles are secreted from the egg to the perivitelline space and transferred to the sperm head membrane, thereby conferring fusion competence to the sperm [37]. However, this experiment could not be reproduced in some independent laboratories and still causes some controversy.

Even though the exact function of CD9 in complex attachment/fusion machinery on the egg is not fully understood, it presents one of the best-investigated factors and is a starting point for many other hypotheses. There is no evidence for an exact binding partner, as the interaction with IZUMO1, however tempting, has not been proven. The exact role of the protein seems to be in organizing the multiprotein complex and the morphology of the membrane required for the fusion.

##### CD81

A second tetraspanin member, CD81, which resembles CD9 in many features, has been proposed to play a role in fusion, as it is expressed on the egg surface and often interacts with CD9 in other cell-cell fusion systems [68]. Deletion of the *Cd81* gene results in a less dramatic reduction in fertility, however, the double knock-out for both CD9 and CD81 are completely infertile [69]. There is even evidence for an extracellular role of CD81, but this has yet to be clarified [28]. The receptor function cannot be excluded from hypotheses, as CD81 works as a receptor for the hepatitis C viral envelope protein [70]. The exact role of this tetraspanin is still under debate, but it is expected to be part of the fusion machinery, with a partly redundant but still important role in the process.

#### 4.2.2. Glycosilphospatidylinositol (GPI) Anchor

There is strong evidence for a specific requirement of GPI-anchored proteins on the egg membrane. At first, GPI-anchored proteins were removed from the egg surface by bacterial-derived PI-PLC (phosphatidylinositol specific phospholipase C), which blocked binding and fusion capability [71]. The findings were then confirmed by producing knock-out mice with deletion in *Pig-a* gene, encoding the first enzyme in the biosynthesis of GPI, which also resulted in an infertile phenotype [72]. The connection between tetraspanins, which forms membrane domains, and lipid rafts, sites that contain the GPI, are now being investigated with the working hypothesis of CD9 and GPI-anchored proteins controlling the signaling pathway induced by adhesion or participating in the appropriate membrane organization [73].

#### 4.2.3. Folate Receptor 4—Juno

The most exciting recent development in the field of gamete fusion biology has been the discovery of the binding partner for IZUMO1, Folate receptor 4 [2]. This GPI-anchored extracellular protein on the oolemal surface has been named Juno, after the Roman goddess of marriage and fertility. Juno is highly expressed on unfertilized eggs and its pre-incubation with a specified antibody potently prevents fertilization. The expression pattern matches to the IZUMO1 binding one on ovulated eggs.

The knockout experiments revealed the absolute infertility of *Juno^−/−^* female mice, while the males remained fertile both *in vivo* and *in vitro*. The females showed normal mating behavior, but *in vitro* experiments revealed the inability of *Juno^−/−^* eggs to fuse with wild type sperm, even though the *zona pellucida* transition was normal.

When the distribution of the Juno protein on the oolema surface, before and after the fertilization, was assessed, it was detected that the Juno antibody signal disappears before the pronuclear stage of the early embryo. The signal has been detected outside of the oolema, by electron microscopy, suggesting vesiculation of the protein into perivitelline space. This rapid shedding of the protein may play an essential role in the prevention of polyspermy in mammals. If sperm was injected into the egg via intracytoplasmic sperm injection (ISCI) rather than undergoing natural fusion, or the egg was parthenogenetically activated, then this shedding of the Juno protein did not occur. 

Monomeric Juno molecules have been found to cluster with each other in the solution to allow direct interaction with IZUMO1. This topology of Juno in the egg’s membrane may be enabled by CD9, which is well known for organizing membrane order. 

Interaction of IZUMO1 and Juno seems to represent a necessary and essential adhesion step rather than the exact fusogenic action. 

#### 4.2.4. SAS1B (Sperm Acrosomal SLLP1 Binding)

When the first binding factor on the sperm, SLLP1 [27], was found, its partner on the oolema was not known. It was suggested that this protein would be confined to the microvilli-rich region of the egg surface. The protein was identified and characterized as SAS1B, specifically oolemal metalloprotease in 2012 [29]. This protease is concentrated in a dome corresponding to the microvillar region and in the perivitelline space, consistently with the presence of CD9 [37]. When stained with a specific antibody, the protease signal co-localizes with the SLLP1 binding sites on the oolema, indicating interaction. Gene knock-out of the protein in mice showed a significantly lowered fertilization rate. SAS1B is the first oocyte specific oolemal metalloprotease, yet to be implicated in gamete binding during fertilization and in partnership with SLLP1, it is believed to be one of the binding factors in the attachment-fusion machinery on the egg surface. 

Whether attachment involves the same molecules that participate in the fusion has not been determined, but the fact that sperm with intact acrosome, unable to fuse with the oolema, still bind to it, supports the concept of an intricate protein complex forming a machinery on both membranes, involving fusion, attachment and associated proteins. We currently have no ability to differentiate between the physiological attachment of sperm ending in fusion from the artificial sticking that is observed in the *in vitro* assay [74]. 

## 5. Conclusions

Fusion is a critical constituent of sexual reproduction, consisting of coordinated steps culminating in the merger of plasmatic membranes. This cytoplasmic union is achieved through gamete interactions, specifically cell adhesion and subsequent membrane fusion of the gamete plasma membranes. All its molecular components, or the exact mechanism in mice, are not yet known, but some players have been found and an overall concept is emerging. 

The main advantage of knowing at least some factors that play a role in the interaction is that we can use this molecule to look for its binding partners, associated molecules or signaling pathways. As IZUMO1 contains an immunoglobulin domain (Ig), which is known for its interaction with various molecules, it was predicted that it binds to a certain receptor on the surface of the egg membrane. This receptor was found to be the Juno molecule. Nevertheless, both of these molecules do not possess many features associated with a fusogenic molecule and most likely they mainly play a role in gamete binding. The Ig domain is known for binding with ligands on other cells (in *trans*-interactions), as well as for interactions with ligands expressed on the same cell (in *cis-*interactions), therefore IZUMO1 can be a binding partner, as well as a membrane-associated protein of the *bona fide* fusogen. The same can be said about Juno, whose exact molecule structure has not yet been fully examined. This undeniably crucial interaction can be therefore used as a starting point for further investigation and a search for the fusogenic mechanism.

Obviously the research for either new molecules or partners of the known ones requires good experimental design to assess the hypotheses. The ongoing hunt for molecules responsible for fusion uses a battery of well established methods, including the use of anti-gamete monoclonal antibodies subsequently tested in *in vitro* sperm-egg binding assays and in *in vitro* fertilization function-blocking experiments. It was in this way that the IZUMO1 protein was first identified. However, shifts in reproduction paradigms and great changes in the overall view over the past several years show us that the most important factor in this convoluted field remains an experimental design and a very careful assessment of the obtained results.

The typical and possibly the most problematic method of gamete interaction experiments represents the *in vitro* sperm-oocyte binding assay [74], which is hard to interpret in a physiologically meaningful manner. Sperm binding prior to fusion is a stepwise process starting with a loose attachment, progressing to a stronger adhesion and potentially leading to fusion. Even sperm unable to membrane merge, as acrosome-intact sperm, still bind to the oolema of *zona*-free eggs, however transiently. Most of the sperm attached in a typical binding assay are unable to process fusion. It is difficult to distinguish between sperm that is merely attached and sperm that is truly bound, and interpretation might be difficult due to various methods of *zona pellucida* removal, which may not be fully removed and sperm can bind to its residues [41,75]. These problems partly explain why so many factors considered important for fusion have been proved dispensable by recent genetic disruption experiments [26,76].

Knock-out experiments have been very popular in the reproduction field lately. A failure to produce any offspring, a phenotype demonstrating an essential role of the factor in question, is a rare outcome, which only male *Izumo1^−/−^* and female *Juno*^−/−^ mice have shown up to date. If the females exhibit subfertile phenotype, it might indicate that the molecule works in redundant ways with complementary molecules. This outcome might be considered as less informative, but the case of CD81 and CD9 shows that even a moderately subfertile phenotype can provide significant insight. CD9-null females have severely reduced fertility [59,60,61,62], whereas CD81-null mice show only a mild decrease in reproduction [77]. Even though CD81 was at first considered a replaceable and not distinctively important factor in reproduction, the phenotype of double knock-out *Cd9*^−/−^/*Cd81*^−/−^ was shown to be completely infertile and proved the importance of assessing subtle changes in the reproductive phenotype for fully understanding the complex machinery of gametes. Similar delicate differences can be seen in the case of β1-integrin, where the possibility of a various mechanism of fertilization of eggs in wild type and β1-integrin knock-out mice has been proposed [40]. The concentration on only binary assessment of fertility (like any pups *versus* no pups) could result in missing out on certain molecules contributing to reproductive success by less robust systems [78].

Owing to problematic complex interpretation of knock-out experiments, other state-of-the-art methods may prove to provide a significant insight into molecular interaction, leading to the formation of zygote, and should be definitely considered in the experimental design. Among these are expression and genomic techniques, proximity ligation assays, techniques for low-affinity extracellular interactions, and many others.

The working hypothesis in the field of gamete interaction is that various proteins in the adhesion/fusion machinery may have various different roles than just *trans*-interactions and adhesion, but also *cis*-interaction regulating membrane morphology, functionality and cellular signaling. It also seems probable that the system is redundant on many levels. This may not only work towards ensuring successful fusion, but also play a role in fine-tuning species-specific adaptations to fertilization. Possible subfunctionalizing among proteins derived from the same gene family, as shown for the *Izumo* genes [79], could prove to be an important factor causing difficulty in our understanding of the course of molecular interactions. The use of others than the model organisms or studies of species-specific fertilization factors in closely related species [80] might shed an unexpected light on this prospect.

This complexity, coupled with a complicated interpretation of the known facts from various types of experiments, makes the field of gamete fusion biology truly demanding and explains why the true basis of the beginning of life, fusion of gametes, remains so poorly understood after so many years of avid research.

## References

[1] Yanagimachi R. (1988). Sperm-egg fusion. Curr. Top. Membr. Transp..

[2] Bianchi E., Doe B., Goulding D., Wright G. (2014). Juno is the egg Izumo receptor and is essential for mammalian fertilization. Nature.

[3] Inoue N., Ikawa M., A. Isotani A., Okabe M. (2005). The immunoglobulin superfamily protein Izumo is required for sperm to fuse with eggs. Nature.

[4] Ellerman D.A., Pei J., Gupta S., Snell W.J., Myles D., Primakoff P. (2009). Izumo is part of a multiprotein family whose members form large complexes on mammalian sperm. Mol. Reprod. Dev..

[5] Jahn R., Lang T., Südhof T.C. (2003). Membrane fusion. Cell.

[6] Stein K.K., Primakoff P., Myles D. (2004). Sperm-egg fusion: Events at the plasma membrane. J. Cell Sci..

[7] Aguilar P.S., Baylies M.K., Fleissner A., Helming L., Inoue N., Podbilewicz B., Wang H., Wong M. (2013). Genetic basis of cell-cell fusion mechanisms. Trend Genet..

[8] Dimitrov D.S. (2004). Virus entry: Molecular mechanisms and biomedical applications. Nat. Rev. Microbiol..

[9] Taylor M.V. (2002). Muscle differentiation: How two cells become one. Curr. Biol..

[10] Oren-Suisse M., Podbilewicz B. (2007). Cell fusion during development. Trends Cell Biol..

[11] Chan D.C., Kim P.S. (1998). HIV entry and its inhibition. Cell.

[12] Harrison S.C. (2008). Viral membrane fusion. Nat. Struct. Mol. Biol..

[13] Weber T., Zemelman B.V., McNew J.A., Westermann B., Gmachl M., Parlati F., Söllner T.H., Rothman J.E. (1998). SNAREpins: Minimal machinery for membrane fusion. Cell.

[14] Rizo J., Rosenmund C. (2008). Synaptic vesicle fusion. Nat. Struct. Mol. Biol..

[15] Dupressoir A., Marceau G., Vernochet C., Bénit L., Kanellopoulos C., Sapin V., Heidmann T. (2005). Syncytin-A and syncytin-B, two fusogenic placenta-specific murine envelope genes of retroviral origin conserved in Muridae. Proc. Natl. Acad. Sci. USA.

[16] Chen A., Leikina E., Melikov K., Podbilewicz B., Kozlov M.M., Chernomordik L.V. (2008). Fusion-pore expansion during syncytium formation is restricted by an actin network. J. Cell Sci..

[17] White J.M., Delos S.E., Brecher M., Schornberg K. (2008). Structures and mechanisms of viral membrane fusion proteins: Multiple variations on a common theme. Crit. Rev. Biochem. Mol. Biol..

[18] Zuccotti M., Piccinelli A., Rossi P.G., Garagna S., Redi C.A. (1995). Chromatin organization during mouse oocyte growth. Mol. Reprod. Dev..

[19] Tash J.S., Means A.R. (1983). Cyclic adenosine 3',5' monophosphate, calcium and protein phosphorylation in flagellar motility. Biol. Reprod..

[20] Sebkova N., Ded L., Vesela K., Dvorakova-Hortova K. (2014). Progress of sperm IZUMO1 relocation during spontaneous acrosome reaction. Reproduction.

[21] Jin M., Fujiwara E., Kakiuchi Y., Okabe M., Satouh Y., Baba S.A., Chiba K., Hirohashi N. (2011). Most fertilizing mouse spermatozoa begin their acrosome reaction before contact with the zona pellucida during *in vitro* fertilization. Proc. Natl Acad. Sci. USA.

[22] Yanagimachi R., Noda Y.D. (1970). Physiological changes in the post-nuclear cap region of mammalian spermatozoa: A necessary preliminary to the membrane fusion between sperm and egg cells. J. Ultrastruct. Res..

[23] Johnson M.H., Eager D., Muggleton-Harris A., Grave H.M. (1975). Mosaicism in organisation concanavalin A receptors on surface membrane of mouse egg. Nature.

[24] Ebensperger C., Barros C. (1984). Changes at the hamster oocyte surface from the germinal vesicle stage to ovulation. Gamete Res..

[25] Primakoff P., Myles D.G. (2007). Cell-cell membrane fusion during mammalian fertilization. FEBS Lett..

[26] He Z.Y., Brakebusch C., Fässler R., Kreidberg J.A., Primakoff P., Myles D.G. (2003). None of the integrins known to be present on the mouse egg or to be ADAM receptors are essential for sperm-egg binding and fusion. Dev. Biol..

[27] Herrero M.B., Mandal A., Digilio L.C., Coonrod S.A., Maier B., Herr J.C. (2005). Mouse SLLP1, a sperm lysozyme-like protein involved in sperm-egg binding and fertilization. Dev. Biol..

[28] Ohnami N., Nakamura A., Miyado M., Sato M., Kawano N., Yoshida K., Harada Y., Takezawa Y., Kanai S., Ono C. (2012). CD81 and CD9 work independently as extracellular components upon fusion of sperm and Oocyte. Biol. Open.

[29] Sachdev M., Mandal A., Mulders S., Digilio L.C., Panneerdoss S., Suryavathi V., Pires E., Klotz K.L., Hermens L., Herrero M.B. (2012). Oocyte specific oolemmal SAS1B involved in sperm binding through intra-acrosomal SLLP1 during fertilization. Dev. Biol..

[30] Okabe M., Yagasaki M., Oda H., Matzno S., Kohama Y., Mimura T. (1988). Effect of a monoclonal anti-mouse sperm antibody (OBF13) on the interaction of mouse sperm with zona-free mouse and hamster eggs. J. Reprod. Immunol..

[31] Inoue N., Ikawa M., Okabe M. (2011). The mechanism of sperm-egg interaction and the involvement of IZUMO1 in fusion. Asian J. Androl..

[32] Inoue N., Hamada D., Kamikubo H., Hirata K., Kataoka M., Yamamoto M., Ikawa M., Okabe M., Hagihara Y. (2013). Molecular dissection of IZUMO1, a sperm protein essential for sperm-egg fusion. Development.

[33] Inoue N., Kasahara T., Ikawa M., Okabe M. (2010). Identification and disruption of sperm-specific angiotensin converting enzyme-3 (ACE3) in mouse. PLoS One.

[34] Almeida E.A., Huovila A.P., Sutherland A.E., Stephens L.E., Calarco P.G., Shaw L.M., Mercurio A.M., Sonnenberg A., Primakoff P., Myles D.G. (1995). Mouse egg integrin α6β1 functions as a sperm receptor. Cell.

[35] Miller B.J., Georges-Labouesse E., Primakoff P., Myles D.G. (2000). Normalfertilizationoccurs with eggslacking the integrinalpha6beta1 and is CD9-dependent. J. Cell Biol..

[36] Barraud-Lange V., Naud-Barriant N., Saffar L., Gattegno L., Ducot B., Drillet A.S., Bomsel M., Wolf J.P., Ziyyat A. (2007). Alpha6beta1 integrin expressed by sperm is determinant in mouse fertilization. BMC Dev. Biol..

[37] Miyado K., Yoshida K., Yamagata K., Sakakibara K., Okabe M., Wang X., Miyamoto K., Akutsu H., Kondo T., Takahashi Y. (2008). The fusing ability of sperm is bestowed by CD9-containing vesicles released from eggs in mice. Proc. Natl Acad. Sci. USA.

[38] Chen H., Sampson N.S. (1999). Mediation of sperm-egg fusion: Evidence that mouse egg α6β1 integrin is the receptor for sperm fertilinbeta. Chem. Biol..

[39] Evans J.P. (2001). Fertilin β and other ADAMs as integrin ligands: Insights into cell adhesion and fertilization. Bioessay.

[40] Baessler K.A., Lee Y., Sampson N.S. (2009). β1 integrin is an adhesion protein for sperm binding to eggs. ACS Chem. Biol..

[41] Evans J.P., Kopf G.S., Schultz R.M. (1997). Characterization of the binding of recombinant mouse sperm fertilin β subunit to mouse eggs: Evidence for adhesive activity via an egg β_1_integrin-mediated interaction. Dev. Biol..

[42] Cho C., Bunch D.O., Faure J.E., Goulding E.H., Eddy E.M., Primakoff P., Myles D.G. (1998). Fertilizationdefects in sperm from mice lacking fertilin β. Science.

[43] Bigler D., Takahashi Y., Chen M.S., Almeida E.A., Osbourne L., White J.M. (2000). Sequence-specific interaction between the disintegrin domain of mouse ADAM2 (fertilin β) and murine eggs. Role of the α_6_ integrin subunit. J. Biol. Chem..

[44] Yamaguchi R., Muro Y., Isotani A., Tokuhiro K., Takumi K., Adham I., Ikawa M., Okabe M. (2009). Disruption of ADAM3 impairs the migration of sperm into oviduct in mouse. Biol. Reprod..

[45] Nishimura H., Cho C., Brandiforte D.R., Myles D.G., Primakoff P. (2001). Analysis of loss of adhesive function in sperm lacking cyritestin or fertilin beta. Dev. Biol..

[46] Mizuno M., Harris C.L., Johnson P.M., Morgan B.P. (2004). Rat membrane cofactor protein (MCP; CD46) is expressed only in the acrosome of developing and mature spermatozoa and mediates binding to immobilized activated C3. Biol. Reprod..

[47] Taylor C.T., Biljan M.M., Kingland C.R., Johnson P.M. (1994). Inhibition of human spermatozoon-oocyte interaction *in vitro* by monoclonal antibodies to CD46 (membrane cofactor protein). Hum. Reprod..

[48] Lozahic S., Christiansen D., Manié S., Gerlier D., Billard M., Boucheix C., Rubinstein E. (2000). CD46 (membrane cofactor protein) associates with multiple beta1 integrins and tetraspa. Eur. J. Immunol..

[49] Inoue N., Ikawa M., Nakanishi T., Matsumoto M., Nomura M., Seya T., Okabe M. (2003). Disruption of mouse CD46 causes an accelerated spontaneous acrosome reaction in sperm. Mol. Cell. Biol..

[50] Johnson P.M., Clift L.E., Andrlíková P., Jursová M., Flanagan B.F., Cummerson J.A., Stopka P., Dvořáková-Hortová K. (2007). Rapid sperm acrosome reaction in the absence of acrosomal CD46 expression in promiscuous field mice (Apodemus). Reproduction.

[51] Stipp C.S., Kolesnikova T.V., Hemler M.E. (2003). Functional domains in tetraspanin proteins. Trends Biochem. Sci..

[52] Claas C., Stipp C.S., Hemler M.E. (2001). Evaluation of prototype transmembrane 4 superfamily protein complexes and their relation to lipid rafts. J. Biol. Chem..

[53] Zoller M. (2009). Tetraspanins: Push and pull in suppressing and promoting metastasis. Nat. Rev. Cancer.

[54] Farquhar M.J., Harris H.J., McKeating J.A. (2001). Hepatitis C virus entry and the tetraspanin CD81. Biochem. Soc. Trans..

[55] Tachibana I., Hemler M.E. (1999). Role of transmembrane 4 superfamily (TM4SF) proteins CD9 and CD81 in muscle cell fusion and myotube maintenance. J. Cell Biol..

[56] Takeda Y., Tachibana I., Miyado K., Kobayashi M., Miyazaki T., Funakoshi T., Kimura H., Yamane H., Saito Y., Goto H. (2003). Tetraspanins CD9 and CD81 function to prevent the fusion of mononuclear phagocytes. J. Cell Biol..

[57] Glazar A.I., Evans J.P. (2009). Immunoglobulin superfamily member IgSF8 (EWI-2) and CD9 in fertilisation: Evidence of distinct functions for CD9 and a CD9-associated protein in mammalian sperm-egg interaction. Reprod. Fertil. Dev..

[58] Le Naour F., André M., Boucheix C., Rubinstein E. (2006). Membrane microdomains and proteomics: Lessons from tetraspanin microdomains and comparison with lipid rafts. Proteomics.

[59] Chen M.S., Tung K.S., Coonrod S.A., Takahashi Y., Bigler D., Chang A., Yamashita Y., Kincade P.W., Herr J.C., White J.M. (1999). Role of the integrin-associated protein CD9 in binding between sperm ADAM 2 and the egg integrin α6β1: Implications for murine fertilization. Proc. Natl. Acad. Sci. USA.

[60] Miyado K., Yamada G., Yamada S., Hasuwa H., Nakamura Y., Ryu F., Suzuki K., Kosai K., Inoue K., Ogura A. (2000). Requirement of CD9 on the egg plasma membrane for fertilization. Science.

[61] Le Naour F., Rubinstein E., Jasmin C., Prenant M., Boucheix C. (2000). Severely reduced female fertility in CD9-deficient mice. Science.

[62] Kaji K., Oda S., Shikano T., Ohnuki T., Uematsu Y., Sakagami J., Tada N., Miyazaki S., Kudo A. (2000). The gamete fusion process is defective in eggs of Cd9-deficient mice. Nat. Genet..

[63] Waterhouse R., Ha C., Dveksler G.S. (2002). Murine CD9 is the receptor for pregnancy-specific glycoprotein 17. J. Exp. Med..

[64] Ellerman D.A., Ha C., Primakoff P., Myles D.G., Dveksler G.S. (2003). Direct binding of the ligand PSG17 to CD9 requires a CD9 site essential for sperm-egg fusion. Mol. Biol. Cell..

[65] Jégou A., Ziyyat A., Barraud-Lange V., Perez E., Wolf J.P., Pincet F., Gourier C. (2011). CD9 tetraspanin generates fusion competent sites on the egg membrane for mammalian fertilization. Proc. Natl. Acad. Sci. USA.

[66] Zhu X., Evans J.P. (2002). Analysis of the roles of RGD-binding integrins, α_4_/α_9_ integrins, α_6_ integrins, and CD9 in the interaction of the fertilin β (ADAM2) disintegrin domain with the mouse egg membrane. Biol. Reprod..

[67] Runge K.E., Evans J.E., He Z.Y., Gupta S., McDonald K.L., Stahlberg H., Primakoff P., Myles D.G. (2007). Oocyte CD9 is enriched on the microvillar membrane and required for normal microvillar shape and distribution. Dev. Biol..

[68] Horváth G., Serru V., Clay D., Billard M., Boucheix C., Rubinstein E. (1998). CD19 is linked to the integrin-associated tetraspans CD9, CD81, and CD82. J. Biol. Chem..

[69] Rubinstein E., Ziyyat A., Prenant M., Wrobel E., Wolf J.P., Levy S., le Naour F., Boucheix C. (2006). Reduced fertility of female mice lacking CD81. Dev. Biol..

[70] Pileri P., Uematsu Y., Campagnoli S., Galli G., Falugi F., Petracca R., Weiner A.J., Houghton M., Rosa D., Grandi G. (1998). Binding of hepatitis C virus to human CD81. Science.

[71] Coonrod S.A., Naaby-Hansen S., Shetty J., Shibahara H., Chen M., White J.M., Herr J.C. (1999). Treatment of mouse oocytes with PI-PLC releases 70-kDa (pI 5) and 35- to 45-kDa (pI 5.5) protein clusters from the egg surface and inhibits sperm-oolemma binding and fusion. Dev. Biol..

[72] Alfieri J.A., Martin A.D., Takeda J., Kondoh G., Myles D.G., Primakoff P. (2003). Infertility in female mice with an oocyte-specific knockout of GPI-anchored proteins. J. Cell Sci..

[73] Lefèvre B., Wolf J.P., Ziyyat A. (2010). Sperm-egg interaction: Is there a link between tetraspanin(s) and GPI-anchored protein(s)?. Bioessays.

[74] Talbot P., Shur B.D., Myles D.G. (2003). Cell adhesion and fertilization: Steps in oocyte transport, sperm-zona pellucida interactions, and sperm-egg fusion. Biol. Reprod..

[75] Yamagata K., Nakanishi T., Ikawa M., Yamaguchi R., Moss S.B., Okabe M. (2002). Sperm from the calmegin-deficient mouse have normal abilities for binding and fusion to the egg plasma membrane. Dev. Biol..

[76] Inoue N., Nishikawa T., Ikawa M., Okabe M. (2012). Tetraspanin-interacting protein IGSF8 is dispensable for mouse fertility. Fertil. Steril..

[77] Kaji K., Oda S., Miyazaki S., Kudo A. (2002). Infertility of CD9-deficient mouse eggs is reversed by mouse CD9, human CD9, or mouse CD81; polyadenylated mRNA injection developed for molecular analysis of sperm-egg fusion. Dev. Biol..

[78] Evans J.P. (2012). Sperm-egg interaction. Annu. Rev. Physiol..

[79] Grayson P., Civetta A. (2012). Positive selection and the evolution of izumo genes in mammals. Int. J. Evol. Biol..

[80] Grayson P., Civetta A. (2013). Positive selection in the adhesion domain of Mus sperm Adam genes through gene duplication and function-driven gene complex formations. BMC Evol. Biol..

